# Designing health professional education curricula using systems thinking perspectives

**DOI:** 10.1186/s12909-020-02442-5

**Published:** 2021-01-06

**Authors:** Priya Khanna, Chris Roberts, Andrew Stuart Lane

**Affiliations:** Sydney Medical School, University of Sydney, Sydney, New South Wales Australia

**Keywords:** Curriculum, Educational design, Education, Systems thinking, Complexity

## Abstract

**Background:**

Medical students navigate complex personal learning pathways from entry into medical school, through an educational program, and into life-long practice. However, many stakeholders have called for substantive reforms in contemporary curricula, citing concerns about the lack of key abilities amongst newly graduated doctors to work in complex healthcare environments. Despite the need for educators to focus on curricula design, there is a paucity of overarching perspectives that allow synthesis of the various curricular elements in a way that lends meaningfulness and appreciation to the students in terms of navigating the immediate program requirements and beyond. Without such guidance, educators risk creating fragmented program designs that can lead to both unintended and unactionable outcomes for students as well as curriculum designers. Using systems thinking, we set out to address this gap by providing an overarching perspective for curriculum designers to appreciate the relationships and the interactions of the various curricular elements that inform and impact student’s preparedness for practice.

**Methods:**

By framing a curriculum as a complex adaptive system, we used soft systems thinking to develop an initial prototype of a conceptual curricular toolkit, underpinned by an appraisal of relevant literature within health professional education and the broader educational context. The prototype was further refined iteratively after critical reflection by the authors with a diverse range of national and international colleagues via posters, short communications, and workshops at several conferences, and through social media.

**Results:**

We describe how the 3P-6Cs toolkit captures a learner’s personal journey through an educational program into a field of practice by logically linking the three key elements: the personal, the program, and the practice. We demonstrate its application in three examples related to contemporary health profession education curricula. These are: creating integrated educational designs to capture students’ developmental continua, conceptualising immersive clinical placements in non-traditional settings, and complexity-consistent evaluation of curricular interventions.

**Conclusion:**

Applying the 3P-6Cs curricular toolkit to problems of curricula (re)design can provide overarching perspectives that enable educators to have a better understanding of how integration of elements within education programs can inform and impact student’s preparation for lifelong practice.

## Background

On their journey through medical school into practice as independent healthcare clinicians, present day students navigate various curricular landscapes that are becoming increasingly progressive and complex. With healthcare practice rapidly evolving to adapt to changing disease patterns and service models, medical curricula have also evolved over time, leading to contemporary models that largely focus on achieving the competencies required to work in healthcare environments [1]. Inspite of the well documented intended learning outcomes within sophisticated blueprints, a lack of key abilities amongst newly graduated medical students still persists [2], leading to *increasing calls for reforms in* medical education programs, particularly in curriculum design [3]. Contemporary curricular reforms, both in undergraduate as well as postgraduate settings, are mostly targeted towards reforming individual elements of a program such as vertical integration of basic and clinical sciences; competency-based learning outcomes, and programmatic and entrustable task-based approaches to assessments. The resultant curricular blueprints, although based on sound educational perspectives to fostering and assessing graduate competencies, are complex to conceptualise and challenging to implement. Without an explicit reference to and inclusion of the ‘big picture’ elements of practice that goes beyond the program they are enrolled into, students may struggle with sense-making in relation to their learning trajectory [4, 5].

In a hypothetical but plausible scenario, a beginning first-year medical student is left in awe as she reads the curriculum handbook of a medical school that has recently renewed its curriculum in the light of contemporary approaches to learning and assessment. Although appreciating the sophisticated design, she does seem lost in the complex labyrinth of educational jargon. The student finds herself trying to decipher the basics: what does she need to learn; what and where are the learning activities to be undertaken, and when and how will she be assessed? The student’s journey of learning, however, does not conclude with graduation but continues as she joins the community of practice of clinicians in the healthcare workforce. A challenging task for the curriculum designers therefore, is to ascertain coherence of various curricular elements while ensuring that their educational utility is appreciated beyond the immediate program requirements by both learners and educators.

A gap, however, persists in relation to overarching curriculum perspectives that allow for synthesis of the various curricular elements in a way that lends coherence and meaningfulness to all stakeholders, particularly students. The paucity of synthetic and overarching curricular perspectives can be inferred from the literature that broadly suggests two key models that underpin medical program curricula: prescriptive and descriptive models [6]. Contemporary approaches to redesigning medical education curricula are largely outcomes-based approaches that emphasise the functional capabilities to be acquired by graduates at the end of a medical program [7]. Such approaches are based on prescriptive models of curriculum as their emphasis is more on the *ends* rather than the *means* of the curricular process [6]. Proponents of prescriptive curricular models, such as competency-based approaches, believe them to be intuitive and promising in mitigating challenges for new graduates in negotiating the contemporary healthcare landscape. Critics, however, have highlighted the risks associated with the outcome-based approaches at the conceptual, assessment, and practical levels [8] including tendencies to reductionism and oversimplification of complex capabilities and critical skills such as professional judgement [9]. Although not as widespread as prescriptive models, there have been attempts towards more descriptive models of curriculum development such as Skilbeck’s situational model [10] and Pinar et al’s curriculum reconceptualization theory, and more recently, the symbiotic curricular model. An example of this is the PRISMS model (product focused’; ‘relevant’; ‘inter-professional’; ‘shorter, smaller’; ‘multi-site’; and ‘symbiotic’) that aims to guide curricular designs in building and reinforcing relationships between medical schools and healthcare services [11]. However, these are not as widespread as the prescriptive models, and they do not inform how different components of the curriculum will work in unison to capture the learning trajectory as the student progresses within a particular program in order to practise in evolving healthcare models and practices [6, 12].

Exploring the natural history of a curriculum from design to implementation can indicate major changes in the original philosophy and rationale, resulting in a fragmented approach, with differing curricular elements being underpinned by differing learning theories [13, 14]. Curricular designs in the present day and age, therefore, need a more holistic approach that takes into account intersections and interrelations between the program-level curricular elements and the larger contexts of practice-level subsystems whilst keeping in view learner’s developmental progression in core capabilities. A coherent and overarching theoretical approach underpinning complex curricular blueprints will ensure the integrity of a whole of curriculum approach [15].

A substantial shift in thinking is therefore required from conceiving of a curriculum as a single mechanical entity that can be ‘fixed’ by attending to individual elements towards understanding the ecology of how various intersecting and interrelating curricular components impact a learner’s trajectory from *being* an individual student to *becoming* a member of a community of practice.

Such a conceptual shift for curriculum designers can be provided by systems thinking. Regarded as a synergistic thinking toolkit, systems thinking aims to understand the interrelationships, dependencies, and interactions shaping the dynamics of the various elements within and across various levels of systems and sub systems [15–17]. It is considered as a highly relevant perspective to understand, predict and improve the capabilities of complex adaptive systems such as a curriculum [18]. In this paper, using systems thinking as a theoretical referent, we aim to provide curriculum designers with a thinking toolkit that enables educators as well as learners in understanding how the various components of a curriculum can be better designed to capture a learners’ journey as they traverse along program-level requirements while keeping in sight preparedness for practice in rapidly evolving complex healthcare settings. By the term ‘thinking toolkit’, we mean a set of ideas, perspectives and approaches, informed by soft systems thinking, that will enable educators to visualise, create, improve, implement, and evaluate new or existing curricular designs.

## Methodology

We developed an initial prototype of an overarching curricular toolkit by undertaking an appraisal of relevant literature in curricular designs for complex systems, especially with reference to using a systems thinking approach. This approach was taken given the authors’ familiarity with this field, and commitment to a curricular design process that avoided the risk of a fragmented approach by focusing on individual curricular subsystems and multiple learning theories [14]. The prototype was further refined iteratively after critical reflection by the authors with a diverse range of national and international colleagues via posters, short communications, and workshops at several conferences, and through social media.

### Conceptual framework

Our framework was guided by various systems thinking perspectives [19–21] wherein interactions between various systems and subsystems can be analysed in terms of boundaries, relationships, and perspectives between and within various systemic elements. By the term ‘system’ we take the view of Checkland, [21] that a system is an adaptive whole that can survive and thrive despite the shocks and alarms of inevitable environmental change. In framing curriculum as an adaptive system, each learning subsystem will be properly linked to others allowing for an appropriate flow of information for self-regulation and adaptation to contextual influences. Only by understanding the ecology of the concepts that constitute the system, can we promote balance between the whole and its parts [19]. This ‘*soft-system’* approach is in contrast to the ‘*hard systems’* approach that assumes a linear representation of the real world, albeit a reductionist one. Our view of systems thinking conforms with a ‘soft systems’ approach as a way of generating both engagement with, and insight about, the real world and allows for the different ways in which the different stakeholders may frame similar curricular issues or problems. Soft system thinking has been regarded as an appropriate approach for high-level structuring of a ‘messy’ real-world problem situation. While the hard system approach informs how a system and its subsystems work, a soft systems approach describes why a system works the way it does [21–24]. It is an holistic approach to understand how various components of a sub-system intersect, interrelate, and interact within the context of larger systems [23, 25].

**Fig. 1:**
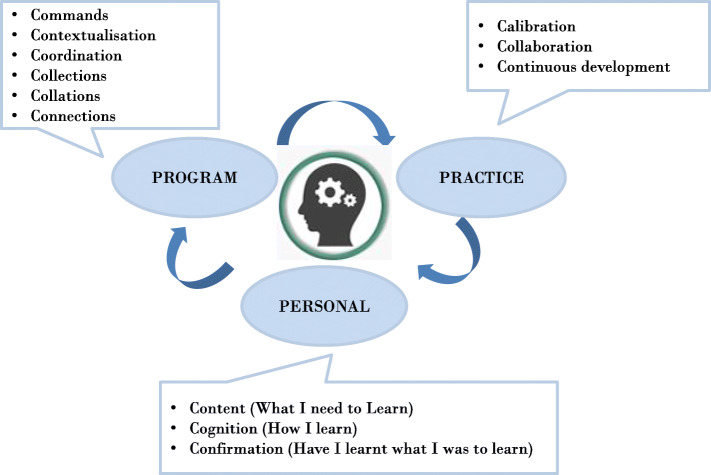
3P-6Cs Systems Thinking Toolkit

With these theoretical underpinnings in mind, we conceptualised a thinking toolkit, ‘3P-6Cs’ (Fig. 1) that illustrates a student’s journey through interactions and intersections of various curricular elements at the personal (P_1_), program (P_2_ with 6Cs), and practice (P_3_) levels. The *personal* describes core considerations of learning at the level of an individual learner; the *program* describes the features or elements of a curriculum, both the explicit and hidden, that a learner navigates through; and the *practice* describes the wider context for learning within the clinical workforce.

Our conceptual toolkit is overarching in the sense that it is akin to an umbrella approach that provides a big picture under which several sub-approaches or models can be sheltered and aligned based on their commonalities [26].

#### The personal level

At the personal level, the student begins her journey at the start of the medical program by reflecting on the core personal considerations of learning - what is to be learnt (Content), how to learn (Cognition), and self- assessing that what should be learnt has been learnt (Confirmation). Confirmation of learning (from self and program assessments) will be a major driver of her learning, but other strategies can foster adaptive skills such as reflexivity and metacognition [18]. At a personal level, there may be negotiation of the boundaries between the designed curriculum with the hidden curriculum. This negotiation will be influenced by students' intrinsic motivations, communications, and their beliefs and attitudes as  they interact with agents such as peers, educators, as well as patients, and agencies such as program-level rules, structures, regulations and policies [27].

#### The program level 6Cs

The program level curricular elements constitute the ‘6Cs’ that define and connect various curricular elements within a curriculum framework. These are commands, contextualisation, coordination, collections, collations, and connections (Table 1). The first three Cs are primarily focused on teaching and learning tasks and outcomes: *Commands* (e.g. learning outcomes as defined by the relevant governing bodies); *Contextualisation* (e.g. learning outcomes, settings and environments) and *Coordination* (integrating learning and teaching and settings and activities). The second three Cs are assessment-focused: *Collections* (marks/scores/narratives), *Collations* (combining differing assessments as per curricular domains or themes or competencies to inform decision-making and progression) and *Connections* (communicating results; reflections on learning and evaluation of curricula).

**Table 1:** The 6Cs of teaching-learning-assessment at the program level

*C1: Commands*	Agencies such as university rules, policies, and procedures; accrediting body’s standards and outcomes; and internship/residency frameworks that command and control the high-level vision, mission, outcomes and practices of the curricular design.
*C2: Contextualisation*	Curricular themes, learning outcomes, objectives, content, learning and teaching methodologies, depth and breadth of clinical exposure directed by various contextual factors such as educators, staff, students, and teaching-learning settings.
*C3: Coordination*	Harmonisation of curricular themes, competencies, and outcomes within activities and assessments enabled by coordination between key agents (educators and staff) and structure (e.g. technological support, curricular policies, rules etc.).
*C4: Collections*	Gathering evidence on students’ developmental progression within a particular year and across all years of a program using a program of assessments.
*C5: Collations*	Capturing students' progression in various competencies using collated data from various assessment points in a way that facilitates triangulation, coherence, consistency, improved educational effect, and holistic judgement of students’ progression into the next level.
*C6: Connections*	Connections occur at multiple levels: connecting students with reflections on their performance, and empowering them to be better prepared for the next training level; connecting the network of stakeholders by using their expertise in making judgements about student’s progressions, and connecting the ‘commands’ of standards for a medical program with those of residency/training outcomes by ensuring students are prepared for practice.

#### The practice level

The practice level elements of the thinking toolkit are focused on the big picture for graduates entering practice as one of a community of health care professionals. As the new graduates transition into their next phase of work integrated learning, our toolkit suggests three key processes: 1) fostering learners to ‘calibrate’ the skills, knowledge and behaviours in accordance with their new workplace requirements, and training programs to meet career intentions 2) the self-regulated and calibrated competencies would be exercised in a learning environment that is highly dependent on ‘*collaboration*’ between and within interprofessional teams, and 3) ensuring ‘*continual development’* of lifelong learning as new graduates move from internship into further specialty training.

As an intern, resident or consultant, a lifelong learner would need to fulfil workplace and program requirements. Therefore, personal and program level 6C curricular elements are also at work at the practice level (given learners will undergo a new training program at this level), albeit the focus will be shifted towards calibrated, collaborative and continuous development of knowledge, skills and behaviours.

## IMPLICATIONS OF THE 3P-6Cs SYSTEMS THINKING FRAMEWORK FOR CURRICULAR DESIGNS

We anticipate that a systems thinking toolkit will provide educators with useful insights when redesigning whole or particular curricular elements. This can be achieved by better understanding of the relationships, boundaries and impacts of personal, program, and practice level elements nested within various curricular subsystems. By explicitly linking various curricular elements and considerations at personal, program, and practice levels, the 3P-6Cs model provides a better understanding of the ecology of curricular elements. The thinking toolkit provides a series of heuristics for educators to address key curricular concerns such as: how to help students make sense of underlying curricular blueprints, and helping them see the big picture beyond the immediate program-level concerns by addressing specific learning outcomes to develop their practice-level capabilities.

To illustrate the utility of our toolkit for health professional educators, we describe applications of the 3P-6Cs systems thinking toolkit in three key areas of curricular design:

1. creating integrated educational designs to capture students’ developmental continua,
2. conceptualising immersive clinical placements in non-traditional settings; and
3. complexity-consistent evaluation of curricular interventions.

1. *Creating a coherent and integrated curricular design to capture students’ developmental continua*

**Fig. 2:**
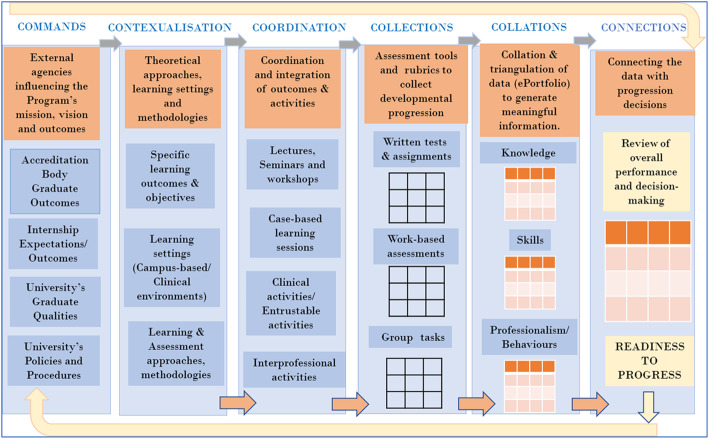
Application of 6Cs to create a coherent and integrated curricular design

Several healthcare professional programs are aspiring towards competency or outcomes-based frameworks that aim to capture the developmental continua of students in milestones or standards of performance in various competencies and sub-competencies [28]. Such a program requires a synthetic and agile design that can meaningfully triangulate complex knowledge, skills and behaviours sampled across various clinical contexts, activities and assessments [15, 22]. An example of the utility of the program level 6Cs to capture students’ progression within a curriculum using a program of learning-teaching-assessments is demonstrated in Fig. 2. Expanding the concept of Bigg’s constructive alignment [29], the program level 6Cs facilitate the following: constructive alignment of external ‘commands’ that direct program-level learning outcomes with ‘contextualisation’ of outcomes and learning methods keeping in view the local flavour in which the program is embedded; and ‘coordination’ of competencies and outcomes with learning-teaching-settings and and learning activities. For example, in our example (Fig. 2) there is a focus on case -based learning. The ‘collection’ of information on students’ progression in various curricular competencies can be meaningfully captured longitudinally using a system of assessment comprising relevant data tools and assessment rubrics (illustrated as matrices for written, work-based and group tasks in Fig. 2). These collections are then meaningfully ‘collated’ within an assessment record, for example, within a portfolio or program of assessments, in our example (Fig. 2) around three vertical themes of knowledge, skills, and professional behaviours. These are communicated to form ‘connections ‘for students, faculty and program designers: for students to connect the gaps in their learning when reflecting on their achievements, and for faculty to reflect on the educational impact of tasks, assessments, and progression decisions. Finally, for program designers to reflect on how well the various elements of the curriculum were constructively aligned.

2. *Conceptualising immersive clinical placements designs in non-traditional settings*

Several medical education programs, as well as other healthcare disciplines, are now offering early and varied clinical immersion by providing placements beyond traditional hospital settings, such as in general practice, ambulatory care, and allied healthcare settings. There is ample and robust evidence for the benefits of early exposure to non-hospital based settings, not only for students in improving a diverse range of skills for the holistic care of patients, but for the medical school in terms of improving graduate outcomes for the program, and establishing a community of diverse preceptors. At the practice level, such immersions can facilitate in bridging the general practitioner-specialist divide as well as the doctor-allied healthcare practitioner divide in providing collaborative patient centred care [30, 31].

**Fig. 3:**
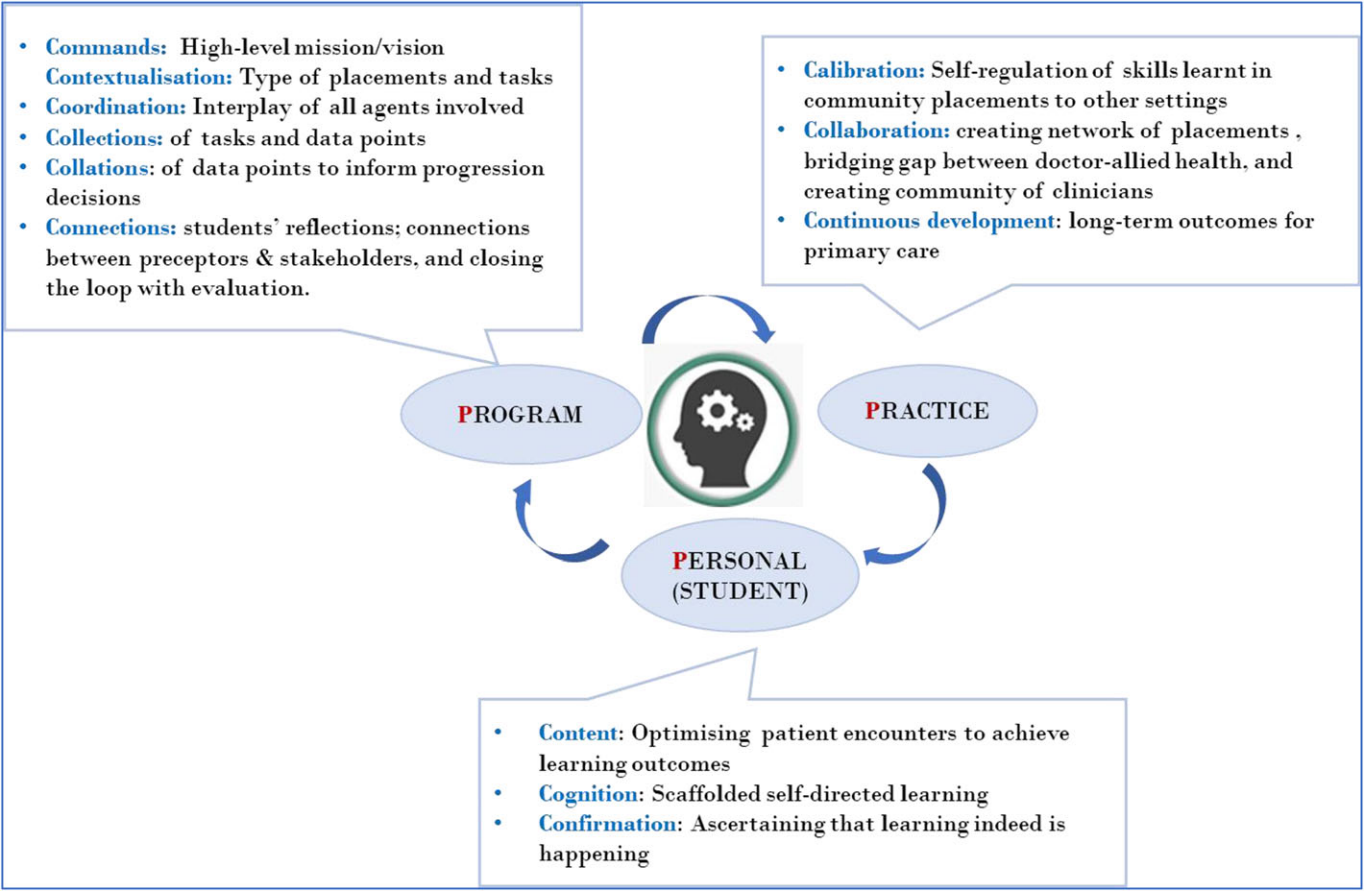
Applying 3P-6Cs in creating coherent mini-curriculum for immersive clinical placements

Keeping in view these benefits of diverse clinical exposure at the personal, program and practice levels, the 3P-6Cs can guide design considerations for an authentic learning task-based curriculum that can bridge the gap between education needs (immediate program requirements) and service needs (creating a community of diverse practices and practitioners) (Fig. 3).

By applying the 3P-6Cs, educators can facilitate in bringing the key stakeholders together in order to workshop the important principles of an immersive clinical placement. This may include the contextualisation of the overarching learning outcomes, collecting observations and feedback of student learning behaviour in the community, developing a community of practice of committed educators, and building capacity in practices offering placements.

3. *Designing complexity-consistent evaluation frameworks*

Curricula are complex adaptive systems and curricular interventions are complex in themselves [3]. Evaluative inquiry into the impact of interventions in such complex systems involves the quality of being systemic, which is not just the property of the system but also of the methodological ‘lenses’ through which one looks into understanding how and why a system works the way it does. For complex interventions, such a systems-based approach can guide research and evaluation paradigms and enable in formulating appropriate program theories to better predict program interventions and outcomes. Such a complexity consistent worldview, as offered by systems thinking, is appropriate to evaluate curricular interventions where we wish to know not only whether a curricular intervention works, but why or why not, for whom, and in what context. Systems thinking seems to be commensurable with theory-driven approaches such as realist evaluation frameworks that are deemed suitable to evaluate health professional education curricular interventions [32]. More specifically in terms of 3P-6Cs, the personal, program, and practice level considerations can help educators collect and utilise rich data on why, where, and for whom the redesigned curriculum is working or not working, identify the underlying mechanisms and propose possible solutions to fix the problems.

With the evolving complexity of healthcare settings and delivery, there is a well acknowledged shift to systems-based practice (‘how can we improve the system of care’). Systems thinking provides a toolkit that fosters the understanding of interdependencies, interactions, and interrelations across and within any system. It is, therefore, the cornerstone of systems-based practice, and has been regarded as one of the core competencies for clinicians [17, 33]. We propose that this toolkit of system thinking has the potential to lay the foundations for a systems-based curriculum that is, a curriculum that better captures interrelations and intersections of a student’s journey towards systems-based practice in the service of patient care.

## Conclusion

Applying the 3P-6Cs curricular toolkit to problems of curricula redesign can provide overarching perspectives that enable educators to have a better understanding of how integration of elements within medical education programs can impact students' preparation for lifelong practice. It aims to capture linkages between various elements of the curricular landscape as a students traverse from the personal to program to practice levels. Based on thinking systemically (thinking in terms of systems) and applying a systems approach to the critical issues of future patient care, we anticipate that the 3P-6Cs will provide a parsimonious yet coherent thinking toolkit to foster better understanding of the relationships, boundaries, perspective and dependencies of various learning subsystems within a curriculum. Our curricular toolkit provides mechanisms for healthcare professional education curricula to evolve, both in their approach and methods, towards improved alignment with individual components of the training continuum, and with the needs of healthcare systems and the patients which the graduates of the program will serve.

## Data Availability

This paper describes the application of systems thinking to curriculum design. It is not an empirical study. There are no data to share.

## Abbreviations

3P- 6Cs: The system thinking toolkit described in this article
Ps: Personal, Program, and Practice
6Cs: Refer to the program level curricular elements of commands, contextualisation, coordination, collections, collations, and connections
